# Synthesis of a Cellulose-Co-AMPS Hydrogel for Personal Hygiene Applications Using Cellulose Extracted from Corncobs

**DOI:** 10.3390/gels7040236

**Published:** 2021-11-27

**Authors:** Haymanot Enawgaw, Tamrat Tesfaye, Kelem Tiessasie Yilma, Derseh Yilie Limeneh

**Affiliations:** 1 Biorefinery Research Center, Ethiopian Institute of Textile and Fashion Technology, Bahir Dar University, Bahir Dar P.O. Box 1037, Ethiopia; haymanot.eng@gmail.com (H.E.); Beteselam2010@gmail.com (K.T.Y.); derseh2003@gmail.com (D.Y.L.); 2 Textile Chemistry Research and Innovation Center, Ethiopian Institute of Textile and Fashion Technology, Bahir Dar University, Bahir Dar P.O. Box 1037, Ethiopia; 3 Textile Production Research and Innovation Center, Ethiopian Institute of Textile and Fashion Technology, Bahir Dar University, Bahir Dar P.O. Box 1037, Ethiopia

**Keywords:** corncobs, cellulose, hydrogel, swelling ratio, personal hygiene

## Abstract

Cellulose-based hydrogels were prepared by the extraction of cellulose from corncobs after the removal of lignin and hemicellulose with the use of alkali–acid treatment. Acrylate-based hydrogels presently available for personal hygiene uses are not biodegradable. In this study, a biodegradable cellulose-co-AMPS personal hygiene hydrogel was synthesized. The hydrogel was synthesized by graft co-polymerization of 2-acrylamido2-methyl propane sulfonic acid onto corncob cellulose by using potassium persulfate (KPS) as an initiator and borax decahydrate (Na_2_B_4_O_7_·10H_2_O) as a cross-linking agent. Structural and functional characteristics of the hydrogel such as swelling measurements, antimicrobial tests, FTIR spectra and thermogravimetric analysis were done. The hydrogel showed an average swelling ratio of 279.6 g/g to water and 83.3 g/g to a urine solution with a 97% gel fraction. The hydrogel displayed no clear inhibition zone and did not support the growth of bacteria, Gram-positive or -negative. The FT-IR spectra of the hydrogel confirmed the grafting of an AMPS co-polymer onto cellulose chains. The thermal properties of the hydrogel showed three-step degradation, with a complete degradation temperature of 575 °C.

## 1. Introduction

Hydrogels are macromolecular gels that are superabsorbent, gained by the chemical stabilization of hydrophilic polymers that can absorb and hold water more than 100 times the mass of dried polymer networks [1,2,3]. Depending on the number of polymer types which hydrogels are composed of, they can be homo-polymeric or co-polymeric [4,5]. They have a cross-linked three-dimensional network that swells, but is not dissolved, in water within a short time [1,5,6,7,8,9,10]. The cross-linking can be due to chemical cross-linking (covalently linked) agents, physical entanglements of polymeric chains or strong secondary bond formation between polymer chains [11,12].

The absorbing, swelling and resistance to dissolution characteristics of hydrogels depend on the hydrophilic functional groups, porosity and size of the structure, as well as the degree of cross-linking in it [4]. These parameters will vary based on the intended application. For example, high-swelling hydrogels are commonly used in agricultural and hygiene products [13]. Smart hydrogels with different responsive moieties for different stimuli, such as temperature, light, pH, magnetic and electrical fields, etc., can be prepared [7].

Hydrogels are widely used for diapers, drug delivery, cosmetics, corneal, contact lenses, skin, implant, ligaments, cartilage, water reservoirs in agriculture, etc. [14,15]. Hydrogels are used for production of hygiene, including disposable diapers and female napkins, as an absorbent core due to their ability to absorb and retain a large amount of secreted fluids, such as urine and blood [7,8,16,17].

Hydrogels are mainly prepared from synthetic polymers, such as polyacrylic acid, polyamides, polyethylene glycol, polymethacrylamide, poly hydroxyethyl methacrylate and polyN-isopropylacrylamide [6,17]. Acrylate-based hydrogels are the most superabsorbent materials that are presently available for use, and are not biodegradable [8]. Many chemicals used in the production of superabsorbent polymers lead to the development of chronic diaper rash and asthma as well as more serious problems, such as male infertility or even testicular cancer. Furthermore, due to the non-biodegradability and toxicity of disposable diapers, they create notable environmental pollution [7,18].

The increasing demand for biocompatible, biodegradable, environmentally friendly and low-cost products lead to the replacement of synthetic polymers with natural ones. Hydrogels prepared from natural polymers such as starch, cellulose, chitosan, gelatin, etc., have benefits in aspects such as biodegradability, non-toxicity, biocompatibility and availability [3,14,15,19,20].

**Figure 1:**
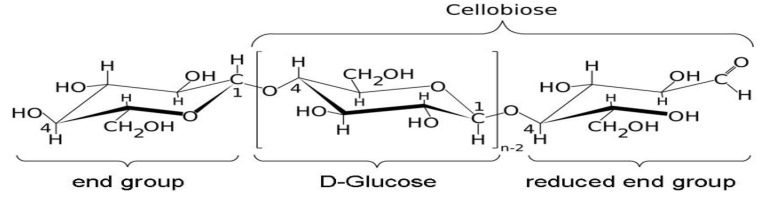
Molecular structure of cellulose.

Hydrogels from cellulose are used mainly for the production of hygienic products and for agriculture purposes, since they are abundant, biodegradable and low-cost [9]. Due to the presence of plentiful hydroxyl groups in cellulose, as shown in Figure 1, cellulose has a big potential for the preparation of bio-based superabsorbent hydrogels [21]. These cellulose hydrogels absorb aqueous solutions through hydrogen bonding with water molecules [16].

Lignocellulosic agricultural residues such as stalks, straws, husks, leaves, etc., can be used as a source for cellulose extraction [22,23]. For instance, corn stover is composed of 15% cob, 51% stalks, 21% leaf blades and 13% husks [24]. These byproducts contain mainly cellulose, hemicellulose and lignin. Beneficiation of these byproducts for the production of biomaterials (plastics, hydrogels, papers, composites, etc.) by extracting these polymers will add value to the corn plant. Conventionally these byproducts are used for animal feeds as well as fuel sources, and some are incinerated [25].

Utilization of corncobs for the production of cellulose-based hydrogels, reducing the percentage of synthetic polymers in synthetic hydrogels and expanding their possible superabsorbent applications in hygiene materials, i.e., diapers. There has been some international research on the utilization of corn byproducts for different applications. Only a few works have been conducted on hydrogel preparation from corncobs. To the knowledge of the authors of this research, no corncob cellulose-co-AMPS hydrogel has been prepared previously.

## 2. Materials and Methods

### 2.1. Materials

Corncobs were collected from local farms from Bahir Dar, Ethiopia. Distilled water, sodium hydroxide (NaOH) supplied from Damao Chemical Reagent Co., Ltd. (Tianjin, China), sodium hypochlorite (NaClO) from Liaocheng Yuanze Chemical Co., Ltd. (Liaocheng, China) and sulfuric acid (H_2_SO_4_) obtained from Xilong Scientific Co., Ltd. (Shenzhen, China) were used for cellulose extraction. 2-Acrylamido-2-methylpropane sulfonic acid (AMPS), potassium persulfate (K_2_S_2_O_8_) and borax decahydrate (Na_2_B_4_O_7_·10H_2_O), supplied from Hefei TNJ Chemical Industry Co., Ltd. (Hefei, China), and ethanol were used for hydrogel preparation. All chemicals used in this research are research-grade.

### 2.2. Methods

#### 2.2.1. Cellulose Extraction from Corncobs

Cellulose was extracted from corncobs by de-lignification, bleaching and acid hydrolysis. De-lignification of corncobs was performed using 10% *w/v* sodium hydroxide (NaOH) at a temperature of 80 °C at a 1:15 solid-to-liquid ratio for 72 min. After de-lignification, double-step bleaching was done using 5% NaClO (*v/v*) at a 1:20 solid-to-liquid ratio at 40 °C for 30 min. Finally, the holocellulose was filtered and washed, followed by acid hydrolysis to remove residual hemicellulose using 10% (*v/v*) sulfuric acid (H_2_SO_4_) at a 1:20 solid-to-liquid ratio at room temperature for 1 h [26,27].

#### 2.2.2. Hydrogel Preparation and Characterization

**Figure 2:**
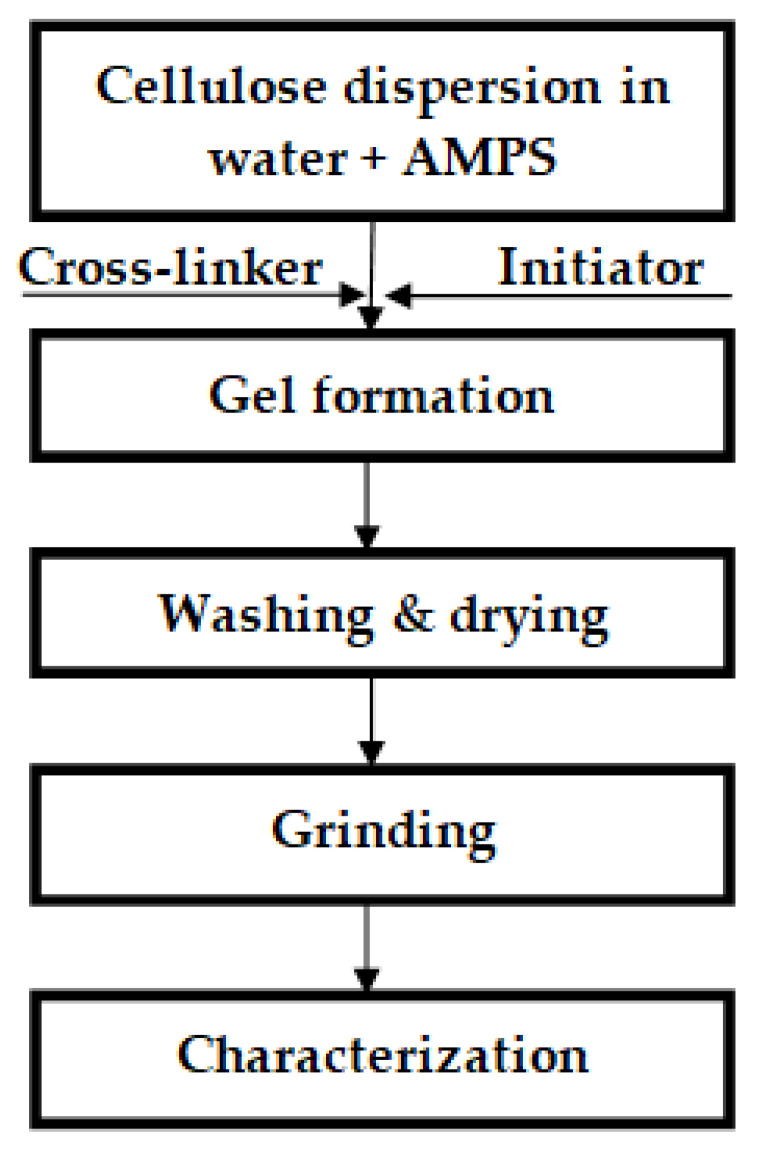
The schematic representation of the hydrogel preparation process.

The general procedure for cellulose-co-AMPS hydrogel formation is indicated in Figure 2. Graft co-polymerization was carried out to prepare the corncob cellulose-co-AMPS hydrogel. A weighted amount of corncob cellulose was first dispersed in water at a 1:10 solid-to-liquid ratio and stirred with a magnetic stirrer until a homogeneous dispersion was obtained; the initiator K_2_S_2_O_8_ (2 wt%) was then added to the dispersed cellulose. AMPS (50 wt%) was added to the dispersion and stirred until the complete dissolution of AMPS. Then, the cross-linker borax decahydrate (5 wt%) was added to the resulting mixture and UV irradiated until the gel was formed. Finally, the resulting hydrogel was washed with 75% ethanol solution and dried in an oven at 60 °C until a constant weight, and then stored in a zipper plastic packaging at a standard laboratory environment for further characterization.

The initiator KPS decomposed forming free sulfate radicals SO_4_^−^ under heating. These sulphate radical anions reacted with hydroxyl radicals, −OH. The hydroxyl radicals then react with the hydrogen atoms from the hydroxyl of C_2_ in the cellulose-chain-forming reactive oxygen O− onto the cellulose chain at C_2_. These macromolecular free radicals can initiate the grafting reaction of AMPS onto the backbone of the corncob cellulose [26].

#### 2.2.3. Characterization of Cellulose-Co-AMPS Hydrogel

***Swelling measurements:*** Of the dried hydrogel, 0.1 g (*W*1) was inserted into a tea bag and immersed in 100 mL of water at room temperature for four hours, till reaching a saturation state of absorption. The swollen hydrogel was then picked up, filtered through a 100-mesh sieve to remove unabsorbed water and weighed as *W*2. Then, the equilibrium swelling ratio (g/g) was determined using Equation (1) [6,8,20]:

(1) $$Swelling\;ratio=\frac{W2-W1}{W1}\;$$

where *W*2 is the weight of the swollen gel and *W*1 is the weight of the dry gel.

***Determination of gel fraction:*** Of the dried hydrogel sample, 0.1 g (*W*1) was immersed in 100 mL of water. For this analysis, the swollen hydrogels were allowed to dry in an oven at 60 °C until the weight was constant. After that, the hydrogel was reweighted and the gel fraction was calculated using Equation (2) [10]:

(2) $$Gel\;fraction\;\%=\frac{W^{\prime }}{W1}$$

where *W*’ refers to the dry hydrogel after the absorbed water is removed and *W*1 is the initial weight of the dry hydrogel.

***Swelling measurements for simulated urine solution:*** By simulating the composition of urine, a solution containing urine components (mainly water, urea, sodium and chloride) was prepared. One hundred milliliters of the solution was prepared by using 95 g of water, 2 g of urea, 1.2 g of sodium chloride and 0.3 g of potassium sulfate. Then, 0.1 g (*W*1) of the dried hydrogel was inserted into a tea bag and immersed in 100 mL of the simulated urine solution at room temperature for four hours, till reaching swelling equilibrium. The swollen hydrogel was then picked up, filtered through a 100-mesh sieve and weighed as *W*2. Then, the equilibrium swelling ratio (g/g) was determined using Equation (3) [6,8,20]:

(3) $$Swelling\;ratio=\frac{W2-W1}{W1}$$

where *W*2 is the weight of the swollen gel and *W*1 is the weight of the dry gel.

***Adsorption kinetics***: The swelling kinetics of the hydrogel upon immersion in liquid (water and simulated urine solution) as a function of time was investigated. Hydrogel samples (0.1 g) were placed into weighed tea bags and immersed completely in 100 mL of solution. The wet bags were then dried in an oven at 105 °C to a constant weight. The absorbency of the hydrogels is then measured at different time intervals (15, 30, 45, 60, 75, 90, 105, 120, 180 and 240 min). Then, the swelling rate was calculated using Equation (4) [20]:

(4) $$SR=\frac{S\left(t+\Delta t\right)-\mathrm{St}}{W1}$$

where *SR* is the swelling ratio, St is the swelling at a time, t, and S(t + Δt) is swelling at a time, t, plus a change in time, Δt.

***Absorbency under load***: This test was conducted by placing a porous filter plate in a Petri dish, and water to the top of the filter plate. A filter paper was placed on the filter plate to be completely wet with the solution. Of the dried hydrogel, 0.1 g was placed into contact with water on the filter screen of a test device. A standard weight to attain a load of 0.3 to 0.7 psi is placed on the top of the hydrogel. After 60 min, the swollen hydrogel was weighted and the absorbency under load was calculated using Equation (5) [20]:

(5) $$AUL=\frac{\mathrm{Weight}\;\mathrm{of}\;\mathrm{swollen}\;\mathrm{gel}-\mathrm{Weight}\;\mathrm{of}\;\mathrm{dried}\;\mathrm{gel}}{\mathrm{Weight}\;\mathrm{of}\;\mathrm{initial}\;\mathrm{hydrogel}}$$

***Antimicrobial effectiveness test:*** An agar disk diffusion test was used to measure the antimicrobial resistance of the cellulose-co-AMPS hydrogel. American Type Culture Collection (ATCC) standard reference strains (*Staphylococcus aureus* ATCC 25923 and *Escherichia coli* ATCC 25922) were used. A concentration of 10^8^ CFU/mL of bacteria suspension was uniformly spread on the agar plate, and the hydrogel sample (100 μL) was placed on the center of the plate and incubated for 24 h at 37 °C. Finally, the diameter of bacterial growth inhibition zones was measured.

***FT-IR analysis:*** Of the dried hydrogel sample, 2 mg was prepared using a KBr powder, and its FT-IR spectra were analyzed by using a (JASCO FT/IR-6600) spectrometer in a range of 4000−400 cm^−1^ and a resolution of 4 cm^−1^, with 400 scans.

***Thermogravimetric analysis (TGA):*** Of the dried hydrogel sample, 2 mg was used for thermal gravity analysis (TG). A thermogravimetric analyzer (thermal balance HCT-1) was used in a temperature range of 30 °C–600 °C at a rate of 20 °C/min, under a 20 mL/min purge flow rate of nitrogen.

## 3. Results and Discussions

### Characterization of Cellulose-Co-Amps Hydrogel

**Figure 3:**
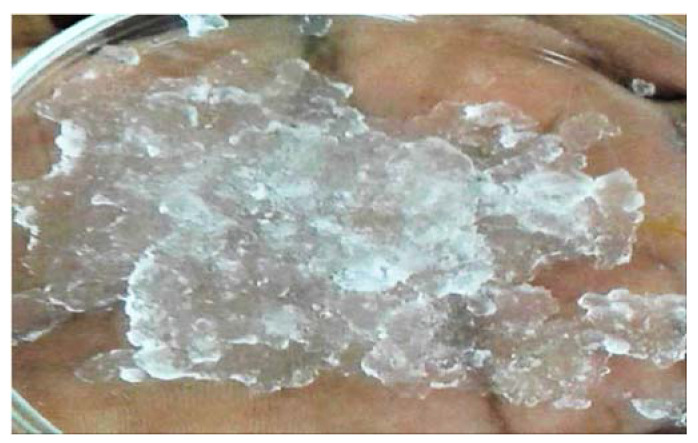
Cellulose-co-AMPS hydrogel after absorption of water.

***Swelling ratio*:** Superabsorbent polymers (SAPs) can absorb and retain large amounts of water or aqueous solution relative to their initial mass. The absorbing capacity of these materials in percentage can reach 1000–100,000% (10–1000 g/g) [28]. The swelling behavior of the corncob cellulose-co-AMPS hydrogel showed a higher swelling capacity. Initially, the hydrogel had a white color, but through water absorption it became a transparent and large gel due to swelling, as can be seen in Figure 3.

The swelling ratio of the corncob cellulose-co-AMPS hydrogel was compared with a commercial superabsorbent polymer (SAP), four replications of each. Surprisingly, the swelling ratio of hydrogels from a mixture of corncob cellulose-co-AMPS is higher than the commercial SAP. This is due to the hydrophilic nature of cellulose, because of its plentiful hydroxyl groups. The swelling ratio of the corncob cellulose-co-AMPS hydrogel was found to be 279.6 g/g (27,960%), in which 1 g of the dried hydrogel sample weighed 279.6 g after water absorption, while the commercial SAP had a 193.8 g/g swelling ratio (19,380%). This is mainly attributed to the additional hydrophilic hydroxyl groups of cellulose in addition to acrylamides. The hydrogel showed higher water absorption compared to the swelling ratio of 50 g/g (5000%) for a wheat straw-based hydrogel reported by [22].

Cheng et al. prepared a corn straw-co-AMPS-co-AA superabsorbent hydrogel and found an optimum water absorbency of 332 g/g of the hydrogels (Cheng et al., 2015). Wang et al. also prepared a superabsorbent hydrogel (CSC-g-AA/APP, CSC-g-AA/PVA-APP) from corn stalk cellulose and found a water absorbency of 262.8 and 303.2 g/g, respectively [3]. Therefore, the swelling ratio of the corncob cellulose-co-AMPS hydrogel in this work is comparable with previous works.

***Determination of gel fraction*:** The gel fraction indicates the number of cross-links formed in the hydrogel structure, that is, the higher the gel fraction the more the hydrogel is cross-linked [27]. Four trials were done, and an average gel fraction of 97% was found for the corncob cellulose-co-AMPS hydrogel. This is due to the removal of uncross-linked structures. [27] found a maximum gel fraction of 99% for cellulose-based hydrogels.

***Swelling measurements for simulated baby urine solution*:** The swelling ratio of the cellulose-co-AMPS hydrogel under absorption of the urine solution was 83.3 g/g (8330%), which is lower than the swelling ratio of the hydrogel to water (279.6 g/g). The reduction in absorption of the hydrogel for the urine solution is due to a change in the composition of the aqueous solution because of additional components in urine (urea, sodium chloride and potassium sulfate) in addition to water.

Hydrogels absorb aqueous solutions through hydrogen bonding with water molecules. Their ability to absorb water is a factor in the ionic concentration of the aqueous solution. In water containing salts and minerals, the absorption potential will vary. This polymer can absorb from up to 500 to 800 times its weight in distilled water, up to 300 times in tap water and from 50 to 60 times in 0.9% NaCl solution. In an 8% CaCl solution swelling is reduced by a factor of ten. The presence of valence cations in the solution will hinder the polymer’s ability to bond with a water molecule [29].

**Figure 4:**
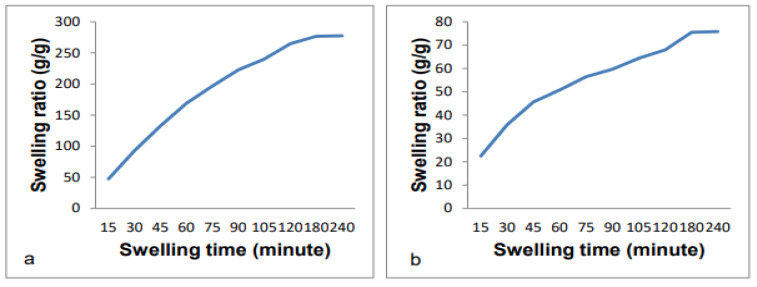
The absorption kinetics of the cellulose-co-AMPS hydrogel under (**a**) water and (**b**) the urine solution.

***Adsorption kinetics***: The adsorption rate of the cellulose-co-AMPS hydrogel was determined both to water and the urine solution (Figure 4). The swelling rate is determined by the size of the initial hydrogel, in which the larger size takes a long time to become completely swollen and reach a saturated level [8]. The swelling kinetics to water showed a swelling ratio of 47.4 g/g at a rate of 3.16 g/min in the first 15 min. The swelling ratio increased to 92.7 g/g for the next 15 min at a rate of 3.02 g/min, and to 132.5 g/g at a rate of 2.65 g/min at a swelling time of 45 min. The swelling ratio reached 168.8 g/g at 2.42 g/min at a time of 60 min. As the swelling time goes to 90 min, the swelling ratio increased to 222.5 g/g, but with a decreased absorption rate of 1.79 g/min. After the second hour (120 min), the swelling was 264.6 g/g with a 1.4 g/min absorption rate. The swelling rate decreased to 0.20 and to 0.01 g/min after the third and fourth hour, respectively.

Due to the constraints discussed in the preceding section, the adsorption rate of cellulose-co-AMPS to the urine solution is again lower when compared to water. The absorption to the urine solution was 22.46 g/g at 1.49 g/min, 35.86 g/g at 0.89 g/min, 45.66 g/g at 0.65 g/min, 50.76 g/g at 0.34 g/min, 56.49 g/g at 0.38 g/min and 59.65 g/min at 0.21 g/min after swelling times of 15, 30, 45, 60, 75 and 90 min, respectively. At a swelling time of 120 min, the swelling reached 67.97 g/g. The swelling rate decreased to 0.12 g/min and 0.006 g/min at a swelling time of 180 and 240 min, respectively.

***Absorbency under load***: The absorbency under load of the cellulose-co-AMPS hydrogel was determined at minimum (60 g/in^2^), medium (120 g/in^2^) and maximum (240 g/in^2^) load. The absorbance of the hydrogel at different loads was found to be 135 g/g, 94 g/g and 63 g/g, respectively. The values indicated that the application of load on the hydrogel reduced its swelling and absorption ability. This value is lower than the swelling ratio of the hydrogel without the load. This is due to the applied load restricting the adsorption of sufficient water and swelling of the hydrogel.

***Antimicrobial effectiveness test*:** The hydrogels to be used for diapers should have resistance to infections caused by microorganisms. Even though the hydrogels produced from cellulose have numerous advantages, they lack antibacterial activity, as cellulose does not have any antibacterial activity [30]. To overcome the aforementioned problems of cellulose-based hydrogels, cellulose is chemically modified by integrating some functional groups to make cellulose resistant to bacteria. The graft co-polymerization process has been frequently used in the production of cellulose-based hydrogels with co-polymers, mainly acrylic acid and acrylamide [30]. The larger the zone of inhibition the more resistance the material is to bacteria [30].

**Figure 5:**
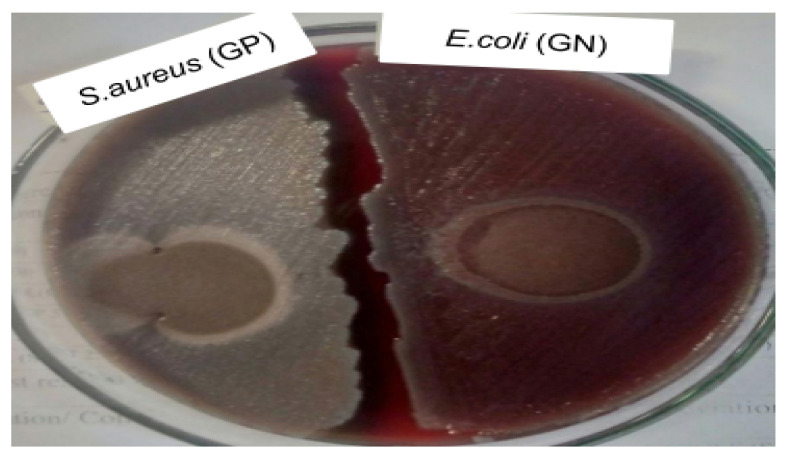
The inhibition zone of the cellulose-co-AMPS hydrogel for Gram-positive and -negative bacteria.

As shown in Figure 5, the cellulose-co-AMPS hydrogel showed no clear inhibition zone, as well as not supporting the growth of bacteria, both Gram-positive and -negative. The antimicrobial groups present in the AMPS polymer (i.e., acrylamide groups) prevent the hydrogel from being attacked by surrounding bacteria. The addition of AA and the AMPS co-polymer to the hydrogel leads to a reduced pH value, which causes stress on the bacterial cells, disrupting the cells’ homeostasis [31,32] prepared carboxymethyl cellulose-g-poly (acrylamide-co-acryl amido-2-methyl-1-propane sulfonic acid) hydrogels. Their sample showed the lowest efficiency as an antimicrobial agent, and they improved the antimicrobial activity of the hydrogel by the addition of silver nanoparticles [32].

**Figure 6:**
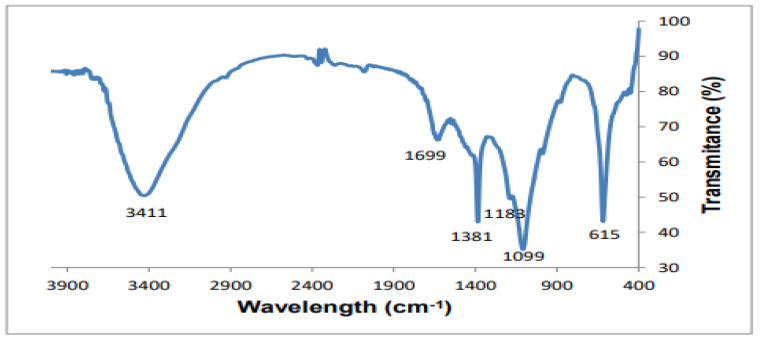
The FT-IR spectra of a cellulose-co-AMPS hydrogel.

***FT-IR analysis:***Figure 6 shows the FT-IR analysis for the cellulose-co-AMPS hydrogel. The transmittance at 3411 cm^−1^ represents the –OH group present in cellulose, and –OH and N– H groups in AMPS [29,33,34]. FT-IR analysis of the cellulose-co-AMPS hydrogel confirmed that AMPS was successfully grafted onto the corncob cellulose. The bands which appeared at 1699 cm^−1^ were characterized to the C=O for AMPS and cellulose [29,33]. The stretching vibration peaks observed at 1612 cm^−1^ were attributed to the C=C backbone present in AMPS polymer. Furthermore, the bands observed at 1381 cm^−1^ and 615 cm^−1^ assigned as the asymmetric stretching of S=O and S-C bonds, respectively, reveal the integration of AMPS into the hydrogel [29]. The band at 1183 cm^−1^ in the hydrogel is attributed to the C-N stretching vibration of amines present in the AMPS backbone.

**Figure 7:**
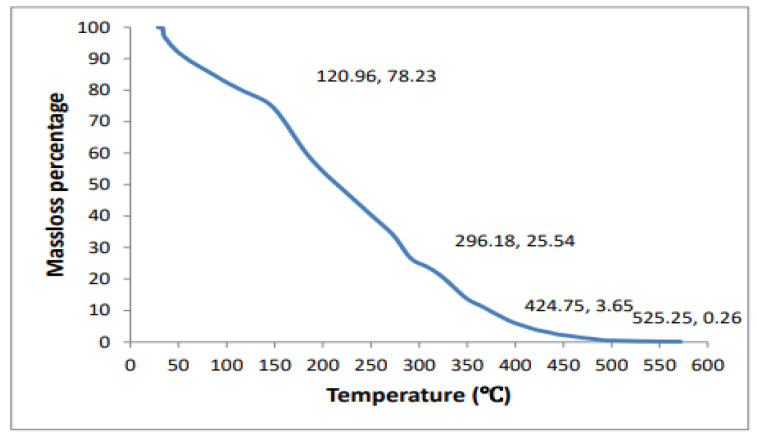
The thermogravimetric analysis of the cellulose-co-AMPS hydrogel.

***Thermogravimetric analysis (TGA)***: Three stages of thermal degradation were observed for the cellulose-co-AMPS hydrogel, as shown in Figure 7. The weight loss observed in the first stage, up to 120 °C, was due to the evaporation of adsorbed water in the hydrogel. The weight loss in the second stage, 120 to 425 °C, was mainly because of the decomposition of the cellulose chain, the dehydration of anhydride due to adjacent carboxyls among polymer backbones and the decarboxylation reaction between carboxyls, which destroyed the cross-linked network structure and polymer chain [31]. The third step of hydrogel degradation, between 425 and 575 °C, is due to the oxidation of charred products [6]. This indicates the high thermal stability of the cellulose-co-AMPS hydrogel due to the cross-linked polymeric network structure.

## 4. Conclusions

Hydrogels prepared from natural polymers have advantages, such as non-toxicity, biodegradability, biocompatibility and availability over hydrogels from synthetic polymers. The admirable biocompatibility of cellulose has encouraged the large use of cellulose-based personal care products. Cellulosic absorbent materials, such as diapers, paper towels, tampons, panty liners and tissue papers, are used as personal care products. Only a slight amount of research has been done on cellulose-based hydrogel preparation from corncobs. None of these have prepared a hydrogel using AMPS as a co-polymer alone, and used borax decahydrate as a cross-linker for the preparation of a cellulose-based hydrogel.

In this study, the cellulose-co-AMPS hydrogel was prepared by graft co-polymerization of 2- acrylamido2-methylpropanesulfonic acid (AMPS) onto cellulose extracted from corncobs. The prepared hydrogel showed an improved swelling capacity, of 279.6 g/g to water and 83.3 g/g to the urine solution with a 97% gel fraction, compared to commercial SAPs, 193.8 (g/g). The absorbency under load of the hydrogel was 94 g/g at a medium level of load (120 g/in^2^) application. The hydrogel showed a higher absorption rate initially and decreased as time increased, mainly after the third hour. The FT-IR spectra of the hydrogel confirmed the formation of the corncob cellulose-co-AMPS hydrogel by showing the characteristic bands of both corncobs and co-polymer units. The TGA results indicated that the hydrogel showed three-step degradation, with a maximum degradation temperature of 575 °C. The hydrogel does not support the growth of both Gram-positive (*Staphylococcus aureus*) and Gram-negative (*Escherichia coli*) bacteria due to the presence of the AMPS co-polymer. Future work will entail the possible utilization of the produced hydrogel for personal hygiene, agriculture, medical and technical applications. Generally, the use of corncobs as a source of cellulose for the production of cellulose-based hydrogels is promising.

## Data Availability

All data and materials are availed in the manuscript and no additional input is required.
